# Evaluation of the Efficacy and Cross-Protective Immunity of Live-Attenuated Chimeric PCV1-2b Vaccine Against PCV2b and PCV2d Subtype Challenge in Pigs

**DOI:** 10.3389/fmicb.2018.00455

**Published:** 2018-03-15

**Authors:** Changchao Huan, Mingyu Fan, Qingru Cheng, Xiaobo Wang, Qingqing Gao, Wanbin Wang, Song Gao, Xiufan Liu

**Affiliations:** ^1^Key Laboratory of Avian Bioproducts Development, Ministry of Agriculture, Yangzhou, China; ^2^Jiangsu Co-innovation Center for Prevention and Control of Important Animal Infectious Diseases and Zoonoses, College of Veterinary Medicine, Yangzhou University, Yangzhou, China; ^3^Institutes of Agricultural Science and Technology Development, College of Veterinary Medicine, Yangzhou University, Yangzhou, China; ^4^Postgraduates Training Station of Jiangsu Province, Taizhou, China

**Keywords:** live-attenuated chimeric porcine circovirus 1-2b, porcine circovirus 2b, porcine circovirus 2d, cross-protective immunity, vaccine

## Abstract

Porcine circovirus type 2 (PCV2) commercial vaccines are either inactivated PCV2 isolates or subunit vaccine based on the Cap protein of PCV2. Currently, no live-attenuated vaccines are yet available. Although the predominant circulating subtype worldwide is PCV2b, the emerging PCV2d subtype is also increasingly associated with PCV disease. In this study, piglets were inoculated with a live-attenuated chimeric PCV1-2b vaccine before challenged with PCV2b and PCV2d isolates. Thirty-two piglets were randomly divided into seven groups: negative (sham-vaccinated, sham-challenged), VAC+PCV2b (PCV1-2b vaccinated, PCV2b-challenged), VAC+PCV2d (PCV1-2b vaccinated, PCV2d-challenged), CHAL+PCV2b (sham-vaccinated, PCV2b-challenged), CHAL+PCV2d (sham-vaccinated, PCV2d-challenged), CV+PCV2b (commercial-vaccinated, PCV2b-challenged), and CV+PCV2d (commercial-vaccinated, PCV2d-challenged). The results showed that vaccinated challenged groups demonstrated high levels of anti-PCV2 antibody and reduced PCV2b and PCV2d loads both in serum and nasal swabs compared with the challenge-only groups. PCV2 DNA was detected in the superficial inguinal lymph nodes of only one pig in each of the VAC+PCV2b and VAC+PCV2d groups (group mean values, 10^1.81^ and 10^1.77^ genomic copies/g, respectively), which was significantly lower than those in CHAL+PCV2b and CHAL+PCV2d animals (group mean values, 10^11.65^ and 10^10.60^ genomic copies/g, respectively; *P* < 0.01). In addition, PCV2 DNA in each of the VAC+PCV2b and VAC+PCV2d groups was significantly lower than those in CV+PCV2b and CV+PCV2d animals (group mean values, 10^8.47^ and 10^8.34^ genomic copies/g, respectively; *P* < 0.01), indicating that the live-attenuated PCV1-2b vaccine was more effective than commercial vaccine. The live-attenuated PCV1-2b vaccine was effective in reducing PCV2b/PCV2d viremia, shedding, and tissue viral loads in vaccinated challenged pigs compared with challenge-only piglets, indicating that the PCV1-2b prototype vaccine is a good candidate for a live-attenuated vaccine against both PCV2b and PCV2d subtypes. And we first revealed that the live-attenuated PCV1-2b vaccine could protect piglets from challenge with either PCV2b or PCV2d equivalently.

## Introduction

Porcine circovirus type 2 (PCV2) is the primary causative agent of global porcine circovirus disease (PCVD). Porcine circovirus type 1 (PCV1) was discovered as a contaminant of the porcine kidney PK-15 cell line in the mid-1970s and is non-pathogenic in pigs. These viruses share approximately 83% nucleotide sequence identity in ORF1, but only 67% identity in ORF2 (Allan and Ellis, 2000). Research has demonstrated that a novel live-attenuated chimeric porcine circovirus 1-2 (PCV1-2) can induce good protective immunity in pigs, and is regarded as a good vaccine candidate against PCV2 (Beach et al., 2010; Fort et al., 2010; Hemann et al., 2014).

PCV2 is divided into five genotypes, include PCV2a, PCV2b, PCV2c, PCV2d, and PCV2e (Xiao et al., 2015; Davies et al., 2016). PCV2a was the first identified PCV2 genotype and predominated in global pig herds until around 2003. The PCV2 genotype then shifted from PCV2a toward PCV2b and was associated with an apparent increase in severity of PCVD in pig herds (Dupont et al., 2008). PCV2c was discovered only in archived swine samples in Denmark and Brazil (Franzo et al., 2015). PCV2d was first identified in 2002 in China and gradually emerged in essentially all pig herds in North America, South America, Europe, and Asia (Segales, 2015). Moreover, several studies indicate that PCV2d is replacing PCV2a and PCV2b to become the predominant strain in pig populations (Franzo et al., 2016). In recent years, PCV2d has been increasing in South America, Europe, United States, Korea, and China (Xiao et al., 2015; Franzo et al., 2016; Kwon et al., 2017). The emergence of PCV2d has been linked to PCVD outbreaks in PCV2-vaccinated herds with vaccines based on PCV2a strains (Opriessnig et al., 2013; Seo et al., 2014; Ramos et al., 2015), but one study shows that the PCV2a inactivated vaccine was effective in protecting pigs against PCV2d infection (Opriessnig et al., 2017). PCV2 vaccination has been effective in protecting pigs from clinical disease and is used extensively today. The currently available commercial vaccines are either inactivated PCV2 isolates or subunit vaccine based on the Cap protein of PCV2, and no live-attenuated vaccines are available to date.

In this study, piglets were inoculated with a novel live-attenuated chimeric PCV1-2b vaccine and challenged with PCV2b or PCV2d. We then investigated the efficacy and cross-protective immunity raised by this vaccine against PCV2b and PCV2d subtypes in pigs.

## Materials and Methods

### Cells and Viruses

The PK-15 cell line, free of PCV1 and PCV2 contamination, was purchased from the China Institute of Veterinary Drug Control (Beijing, China). The PCV2b (PCV2b/1B/Jiangsu/2012/11/18), PCV2d (PCV2d/Changzhou/JS/20150728CZ), and chimeric PCV1-2b strains were propagated in PK-15 cells as described previously (Xujie et al., 2011; Li et al., 2017). Briefly, the chimeric PCV1-2b was constructed as follows: the PCV1 genome was amplified by PCR and ligated into pSK vector. The PCV1 genome without ORF2 (pSK-PCV1 ΔORF2) was amplified by PCR. ORF2 of PCV2b was ligated into pSK-PCV1 ΔORF2. The PCV1-2 DNA clones were dimerized to product more viruses. We confirm that the PCV1-2b is correctly constructed by sequencing. The PCV2d (PCV2d/Changzhou/JS/20150728CZ) strain used in the study was confirmed to be an authentic PCV2d subtype strain isolated in China by sequencing of the entire viral genome.

### Animals, Housing, and Experimental Design

Thirty-two 3-week-old piglets were bought from one pig farm. They were negative for PCV2, porcine reproductive and respiratory syndrome virus, porcine parvovirus, porcine pseudorabies virus, and classical swine fever virus infections. The piglets were divided randomly into seven groups and kept in seven isolation rooms. Prior to vaccination, the piglets were weighed, and blood samples were taken to confirm negativity for PCV2 antibodies. Pigs in the VAC+PCV2b (PCV1-2b vaccinated, PCV2b-challenged, *n* = 5), VAC+PCV2d (PCV1-2b vaccinated, PCV2d-challenged, *n* = 5), CV+PCV2b (commercial-vaccinated, PCV2b-challenged, *n* = 5), and CV+PCV2d (commercial-vaccinated, PCV2d-challenged, *n* = 5) groups were vaccinated intramuscularly (IM) with 1 mL of the chimeric PCV1-2b virus (10^5.0^ TCID_50_ per pig) or commercial vaccine, and challenged with 2 × 10^4.8^ TCID_50_ wildtype PCV2b or PCV2d virus, respectively (1 mL intranasally and 1 mL IM) at 28 days post-vaccination (DPV). The unvaccinated control pigs were also further divided into two groups [CHAL+PCV2b (sham-vaccinated, PCV2b-challenged), CHAL+PCV2d (sham-vaccinated, PCV2d-challenged)] of five pigs each and similarly inoculated with PCV2b and PCV2d, respectively. The negative (sham-vaccinated, sham-challenged) control group consisting of two piglets was neither vaccinated nor challenged with virus (**Table 1**). At 21 days post challenge (DPC), all pigs were necropsied, and macroscopic and microscopic lesions were compared between the groups. The amount of PCV2 antigen in the lymphoid tissues was determined by immunohistochemistry (IHC).

**Table 1:** Experimental design and the vaccination and challenge status of experimental pigs.

Group	Vaccine	Vaccine dose (TCID_50_)	Vaccination day	Challenge	Challenge dose (TCID_50_)	Challenge day
VAC+PCV2b	PCV1-2b	1 × 10^5.0^	21	PCV2b	2 × 10^4.8^	49
VAC+PCV2d	PCV1-2d	1 × 10^5.0^	21	PCV2d	2 × 10^4.8^	49
CHAL+PCV2b	–	–	–	PCV2b	2 × 10^4.8^	49
CHAL+PCV2d	–	–	–	PCV2d	2 × 10^4.8^	49
CV+PCV2b	CV	1 mL	21	PCV2b	2 × 10^4.8^	49
CV+PCV2d	CV	1 mL	21	PCV2d	2 × 10^4.8^	49
Negative group	–	–	–	–	–	–


### Serology

Serum samples were collected weekly from all pigs after vaccination, and tested using an ORF2-based anti-PCV2 IgG ELISA purchased from MEDIAN. Samples were considered positive if the calculated sample-to-positive (S/P) ratio was 0.4 or greater.

### Quantification of PCV2 and PCV1-2b DNA Loads

DNA was extracted from serum samples and swab suspensions collected at 0, 7, 14, 21, 28, 35, 42, and 49 DPV, and superficial inguinal lymph node (SILN) samples were collected during necropsy. All DNA was used for quantification of the chimeric PCV1-2b and PCV2 genomic DNA copy numbers by real-time PCR assay. The real-time PCR used to detect chimeric PCV1-2b was designed to differentiate PCV1-2 chimeric DNA from both PCV2 and PCV1 DNA (Shen et al., 2010). A selective PCV2 real-time PCR was performed by using a conventional PCR to detect the ORF1 of PCV2 as described previously (Li et al., 2015).

### Necropsy, Macroscopic Lesions, and Histopathology

All pigs were necropsied at 21 DPC. Macroscopic lung lesion and SILN sizes were estimated and recorded. Sections of lung (five sections), heart, tonsils, thymus, liver, spleen, small intestine, colon, kidney, and lymph nodes (tracheobronchial, mediastinal, mesenteric, sub iliac, and superficial inguinal) were collected, fixed in 10% neutral buffered formalin, and processed for histopathological examination. Lymphoid tissues were evaluated for lymphoid depletion (LD) and histiocytic replacement (HR) of lymph node follicles.

### Immunohistochemistry

The PCV2-specific antigen was detected by staining formalin-fixed and paraffin-embedded sections of lymph nodes as described previously (Li et al., 2017). A pig polyclonal antiserum was purchased from VMRD, WA, United States. PCV2 antigen scoring was done by veterinary pathologists in a blinded fashion.

### Fluorescence *in Situ* Hybridization

Superficial inguinal lymph nodes were fixed for 6 h at 4°C, and were embedded in optimal cutting temperature (OCT). Then SILNs were cut into 6 μm sections. The SILN sections were processed as described previously (Aida et al., 2007). The sections were hybridized with 1000 ng/mL 5′-FITC-labeled PCV2b and PCV2d DNA probes for 48 h at 65°C. The nuclei were stained with DAPI for 5 min at room temperature. The fluorophores were visualized with Leica DMi8 confocal microscope with a 10× immersion objective. 5′-FITC-labeled PCV2b and PCV2d DNA probes were prepared by GEFAN BIOTECHNOLOGY (Shanghai, China). The probes which could identify the DNA of PCV2b and PCV2d were 5′-FITC-tcagggcatcggggggaaagggtgaccaactggccccctt.

### Statistical Analysis

All data were analyzed by one-way ANOVA using SPSS statistical analysis software (version 13.0) to calculate statistical significance (*P* < 0.05, significant difference; *P* < 0.01 extremely significant difference). Statistical analysis was performed using Prism v6.0 (GraphPad Software, La Jolla, CA, United States), and expressed as mean values with standard deviations.

## Results

### Clinical Presentation

Clinical disease was not observed in any of the pigs following PCV1-2b vaccination or commercial-vaccine vaccination, and none of the pigs had visible reactions (redness, swelling, and higher temperature) at the vaccination site. The temperature of two pigs in the CHAL+PCV2b and one pig in the CHAL+PCV2d was high (40.0–40.3°C). Prior to challenge, the average daily weight gain (ADWG) ranged from 0.19 to 0.22 kg/day, with no significant differences between the groups. After challenge, the ADWG ranged from 0.24 to 0.29 kg/day, with no significant differences between the groups (**Table 2**).

**Table 2:** Fever and ADWG from vaccination day to necropsy day.

Group	Day with fever (≥40°C)	ADWG	
		
		Vaccination to challenge	Challenge to necropsy
VAC+PCV2b	0/5	0.20 ± 0.11	0.27 ± 0.18
VAC+PCV2d	0/5	0.19 ± 0.06	0.24 ± 0.07
CHAL+PCV2b	2/5	0.22 ± 0.08	0.26 ± 0.09
CHAL+PCV2d	1/5	0.19 ± 0.11	0.24 ± 0.04
CV+PCV2b	0/5	0.22 ± 0.08	0.29 ± 0.10
CV+PCV2d	0/5	0.21 ± 0.09	0.28 ± 0.08
Negative group	0/2	0.20 ± 0.07	0.24 ± 0.09


### Anti-PCV2 Antibody Levels

**FIGURE 1:**
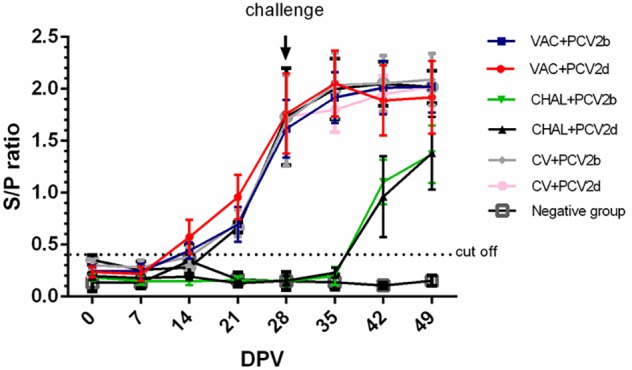
The sample-to-positive (S/P) ratio of each group at different days post vaccination (DPV) by PCV2 ELISA.

At 0 and 7 DPV, all pigs were negative for PCV2-specific antibodies. At 14 DPV, positive PCV2 antibodies were detected in three of five VAC+PCV2b pigs and four of five VAC+PCV2d pigs. At 14 DPV, positive PCV2 antibodies were detected in two of five CV+PCV2b pigs and one of five CV+PCV2d pigs. All pigs were positive at 28 DPV. The mean PCV2 ELISA titers for each group from 0 to 49 DPV are summarized in **Figure 1**. The unvaccinated pigs seroconverted between 7 and 14 days after PCV2 challenge.

### Prevalence and Amount of PCV1-2b DNA in Serum, Nasal Swab, and Fecal Swab Samples

**FIGURE 2:**
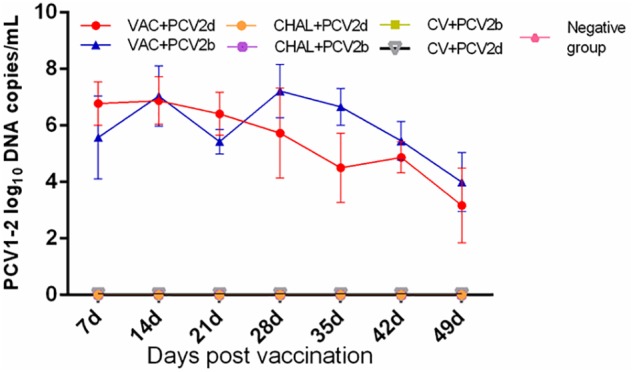
Group mean log_10_ of PCV1-2b genomic copies/mL in serum obtained from pigs at different days post vaccination (DPV).

The PCV1-2b chimeric virus vaccine DNA was detected in the serum of pigs 7 days after vaccination. The amount of serum PCV1-2b DNA peaked at 28 DPV, and was not different among vaccinated groups (*P* > 0.05; **Figure 2**). All other groups were negative for serum PCV1-2b DNA (**Figure 2**). PCV1-2b DNA was detected in nasal and fecal swab samples in the two PCV1-2b vaccinated groups after inoculation. Two of five nasal and fecal swabs were positive for PCV1-2b DNA at 35 DPV and only one of five fecal swabs at 42 and 49 DPV were found in the VAC+PCV2b group (**Table 3**). For the VAC+PCV2d group, one of five nasal swabs were positive at 42 DPV and 49 DPV, and two of five fecal swabs were positive at 35–49 DPV for PCV1-2b DNA (**Table 3**). PCV1-2b DNA was not detected in fecal swabs from the two challenge groups and negative group (**Table 3**).

**Table 3:** Group mean log_10_ of PCV1-2b genomic copies/mL in nasal and fecal swabs obtained from pigs at different days post vaccination.

Group	Sample	Result on trial day/DPV						
		
		7	14	21	28	35	42	49
VAC+PCV2b	Nasal swabs	2/5 (1.72)	2/5 (1.548)	1/5 (0.85)	1/5 (0.80)	2/5 (1.76)	0/5 (0.00)	0/5 (0.00)
	Fecal swabs	2/5 (2.06)	3/5 (3.11)	2/5 (1.80)	4/5 (3.68)	2/5 (1.82)	1/5 (1.77)	1/5 (0.76)
VAC+PCV2d	Nasal swabs	1/5 (0.94)	2/5 (1.78)	2/5 (1.68)	2/5 (1.59)	0/5 (0.00)	1/5 (0.78)	1/5 (1.08)
	Fecal swabs	3/5 (2.89)	4/5 (4.20)	4/5 (3.94)	2/5 (2.00)	2/5 (1.85)	2/5 (1.76)	2/5 (1.68)
CHAL+PCV2b	Nasal swabs	0/5 (0.00)	0/5 (0.00)	0/5 (0.00)	0/5 (0.00)	0/5 (0.00)	0/5 (0.00)	0/5 (0.00)
	Fecal swabs	0/5 (0.00)	0/5 (0.00)	0/5 (0.00)	0/5 (0.00)	0/5 (0.00)	0/5 (0.00)	0/5 (0.00)
CHAL+PCV2d	Nasal swabs	0/5 (0.00)	0/5 (0.00)	0/5 (0.00)	0/5 (0.00)	0/5 (0.00)	0/5 (0.00)	0/5 (0.00)
	Fecal swabs	0/5 (0.00)	0/5 (0.00)	0/5 (0.00)	0/5 (0.00)	0/5 (0.00)	0/5 (0.00)	0/5 (0.00)
CV+PCV2b	Nasal swabs	0/5 (0.00)	0/5 (0.00)	0/5 (0.00)	0/5 (0.00)	0/5 (0.00)	0/5 (0.00)	0/5 (0.00)
	Fecal swabs	0/5 (0.00)	0/5 (0.00)	0/5 (0.00)	0/5 (0.00)	0/5 (0.00)	0/5 (0.00)	0/5 (0.00)
CV+PCV2d	Nasal swabs	0/5 (0.00)	0/5 (0.00)	0/5 (0.00)	0/5 (0.00)	0/5 (0.00)	0/5 (0.00)	0/5 (0.00)
	Fecal swabs	0/5 (0.00)	0/5 (0.00)	0/5 (0.00)	0/5 (0.00)	0/5 (0.00)	0/5 (0.00)	0/5 (0.00)
Negative group	Nasal swabs	0/2 (0.00)	0/2 (0.00)	0/2 (0.00)	0/2 (0.00)	0/2 (0.00)	0/2 (0.00)	0/2 (0.00)
	Fecal swabs	0/2 (0.00)	0/2 (0.00)	0/2 (0.00)	0/2 (0.00)	0/2 (0.00)	0/2 (0.00)	0/2 (0.00)


### Prevalence and Amount of PCV2 DNA in Serum, Nasal Swab, and Fecal Swab Samples

**FIGURE 3:**
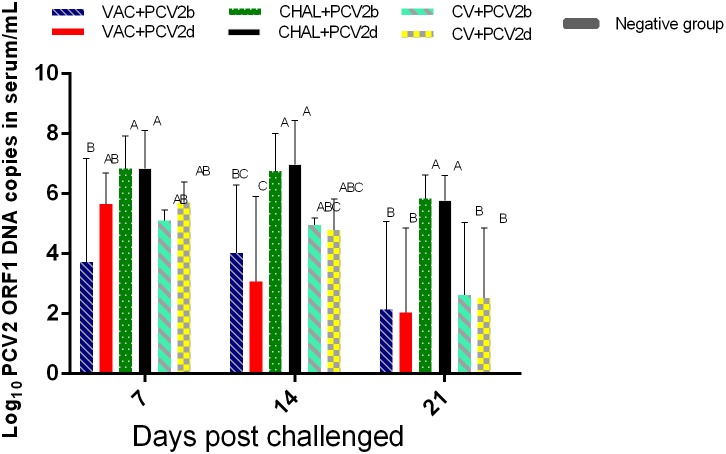
Group mean log_10_ of PCV2 genomic copies/mL in serum obtained from pigs at different days post challenge (DPC). Different superscripts (A, B, C) indicate significant differences among groups (*P* < 0.05).

At 7 DPC, PCV2 DNA in serum from VAC+PCV2b pigs was lower than in the corresponding challenge-only group (*P* < 0.05). At 14 DPC, PCV2 DNA genomic copies were significantly lower in the VAC+PCV2d group compared with the CHAL+PCV2d group (*P* < 0.05). The levels of PCV2 in the serum of four vaccinated groups were also significantly lower than challenge groups at 21 DPC (*P* < 0.05). Furthermore, three pigs in the PCV1-2b vaccinated group, two pigs in commercial-vaccine vaccinated group and negative group demonstrated undetectable serum PCV2 DNA (**Figure 3**).

In the nasal swab samples, PCV2 DNA was detected in two of five pigs from the VAC+PCV2b, VAC+PCV2d, CV+PCV2b, and CV+PCV2d groups at 7 DPC. Following this, the samples from one pig in the PCV1-2b vaccinated group and two pigs in commercial-vaccine vaccinated group were found to be positive for PCV2 DNA at 14 and 21 DPC. For the unvaccinated but challenged groups (CHAL+PCV2b, CHAL+PCV2d), all five of the nasal swab samples were found to carry 10^5.67^–10^6.43^ PCV2 genomic copies from 7 to 21 DPC (**Table 4**). PCV2 DNA was 0 in negative group (**Table 4**).

**Table 4:** Group mean log_10_ of PCV2 genomic copies/mL in nasal and fecal swabs obtained from pigs at different days post challenge (DPC).

Group	Sample	7 DPC	14 DPC	21 DPC
VAC+PCV2b	Nasal swabs	2/5 (1.23)	1/5 (0.75)	1/5 (0.93)
	Fecal swabs	2/5 (1.21)	1/5 (0.96)	0/5 (0.00)
VAC+PCV2d	Nasal swabs	2/5 (1.23)	1/5 (1.18)	1/5 (1.21)
	Fecal swabs	3/5 (3.81)	2/5 (2.51)	2/5 (1.29)
CHAL+PCV2b	Nasal swabs	5/5 (5.75)	5/5 (6.32)	5/5 (5.94)
	Fecal swabs	5/5 (6.06)	5/5 (6.75)	5/5 (6.88)
CHAL+PCV2d	Nasal swabs	5/5 (5.67)	5/5 (6.43)	5/5 (6.18)
	Fecal swabs	5/5 (6.31)	5/5 (6.87)	5/5 (6.56)
CV+PCV2b	Nasal swabs	2/5 (2.08)	2/5 (2.11)	2/5 (1.86)
	Fecal swabs	3/5 (4.21)	3/5 (3.42)	2/5 (2.01)
CV+PCV2d	Nasal swabs	2/5 (2.12)	2/5 (2.18)	2/5 (1.98)
	Fecal swabs	3/5 (4.48)	3/5 (3.71)	2/5 (2.39)
Negative group	Nasal swabs	0/2 (0)	0/2 (0)	0/2 (0)
	Fecal swabs	0/2 (0)	0/2 (0)	0/2 (0)


Regarding the fecal swab samples, PCV2 DNA was detected in two pigs in the VAC+PCV2b group at 7 DPC, but no PCV2 DNA was detected at 21 DPC. For the VAC+PCV2d group, three pigs were also positive for PCV2 DNA at 7 DPC, and two pigs were positive between 14 and 21 DPC. For the CV+PCV2b or CV+PCV2d group, three pigs were also positive for PCV2 DNA at 7 and 14 DPC, and two pigs were positive at 21 DPC. All fecal swabs from the unvaccinated but challenged pigs contained PCV2 DNA between 7 and 21 DPC, carrying 10^6.06^–10^6.88^ PCV2 genomic copies.

### PCV2 and PCV1-2b Viral Load in SILN Tissues

**FIGURE 4:**
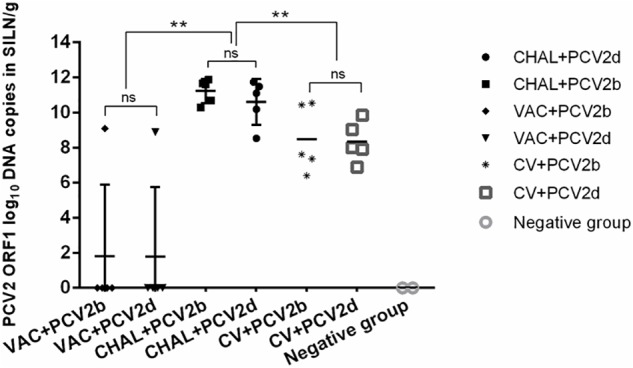
PCV2 DNA loads (copies/g) in SILN tissues collected on the day of necropsy (ns, *P* > 0.05; ^∗∗^*P* < 0.01).

The PCV2 viral loads in SILN tissues after challenge was measured (**Figure 4**). At 21 DPC, only one pig in each of the VAC+PCV2b and VAC+PCV2d groups showed detectable PCV2 viral DNA in its SILN tissues (group mean values, 10^1.81^ and 10^1.77^ genomic copies/g, respectively). The viral load in the SILN tissues of the CV+PCV2b and CV+PCV2d pigs ranged from 10^6.39^ to 10^10.55^ genomic copies/g. The viral load in the SILN tissues of the CHAL+PCV2b and CHAL+PCV2d pigs ranged from 10^8.53^ to 10^11.87^ genomic copies/g. The viral load present in the SILN from each of the vaccinated groups was found to be significantly lower than unvaccinated but challenged pigs at 21 DPC (*P* < 0.01). The viral load in the SILN tissues of negative group was 0 (**Figure 4**).

**FIGURE 5:**
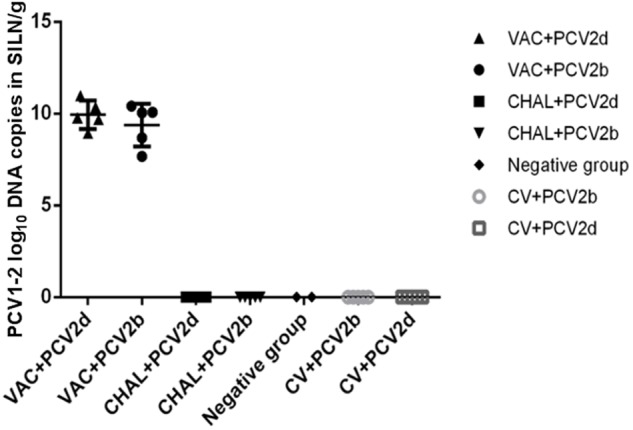
PCV1-2b DNA loads (copies/g) in SILN tissues collected on the day of necropsy.

PCV1-2b DNA was detected in all SILN tissues of PCV1-2b vaccinated pigs at 21 DPC. The group mean values for the VAC+PCV2b and VAC+PCV2d groups were 10^9.37^ and 10^9.94^ genomic copies/g, respectively. There was no statistically significant difference in the PCV1-2b viral load of the SILN tissues between either of the vaccinated groups (*P* > 0.05). Commercial-vaccine vaccinated pigs, unvaccinated pigs and pigs in negative group showed no detectable PCV1-2b DNA in the SILN tissues (**Figure 5**).

### Microscopic Lesions

**FIGURE 6:**
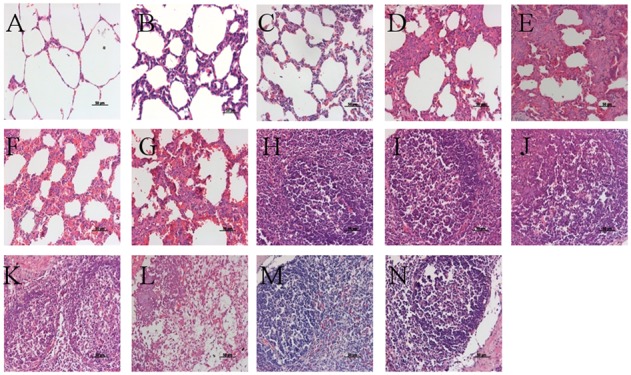
Histopathological lesions in experimental pigs. **(A)** No remarkable microscopic lesions were found in the lungs of the negative group pigs. **(B)** Slight interstitial pneumonia was found in the lung of one of five VAC+PCV2b groups pigs. **(C)** Slight lymphoplasmacytic and histiocytic bronchointerstitial pneumonia was present in the lungs of two of five VAC+PCV2d group pigs. **(D)** Severe lymphoplasmacytic and interstitial pneumonia was found in the lungs of four of five unvaccinated PCV2b-challenged pigs. **(E)** Severe interstitial pneumonia was observed in the lungs of four of five unvaccinated PCV2d-challenged pigs. **(F)** Moderate lymphoplasmacytic and interstitial pneumonia was observed in the lung two of five CV+PCV2b group pigs. **(G)** Moderate lymphoplasmacytic and interstitial pneumonia was observed in the lung two of five CV+PCV2d group pigs. **(H)** No remarkable microscopic lesions were found in the lymph nodes of pigs in the mock group. **(I)** No remarkable microscopic lesions were found in the lymph nodes of VAC+PCV2b group pigs. **(J)** Slight lymphocyte deletion was observed in the lymph node follicles of two of five VAC+PCV2d group pigs. **(K)** Severe macrophage accumulation was found in lymph node follicles of all five unvaccinated PCV2b-challenged pigs. **(L)** Severe lymphoid depletion (LD) was observed in lymph node follicles of four of five unvaccinated PCV2d-challenged pigs. **(M)** Severe eosinophil infiltration in the lymph nodes in one pig of CV+PCV2b group. **(N)** LD in lymph nodes follicles in one pig of CV+PCV2d group.

At 21 DPC, microscopic lesions in various tissues are summarized in **Table 5**. No pig in the VAC+PCV2b group showed obvious microscopic lesions in SILN tissues (**Figure 6I**), and only one pig exhibited the type of slight lung lesions that are characteristic of interstitial pneumonia (**Figure 6B**). In the VAC+PCV2d group, two of five pigs had mild to moderate LD (**Figure 6J**) and one pig had slight HR in SILN tissues, while two exhibited slight interstitial pneumonia in the lung tissue (**Figure 6C**). All pigs in the CHAL+PCV2b group and four of five pigs in the CHAL+PCV2d group exhibited moderate to severe microscopic lesions of SILN tissues (**Figures 6K,L**). Additionally, four of five pigs in both the CHAL+PCV2b and CHAL+PCV2d groups demonstrated moderate to severe interstitial pneumonia in lung tissues (**Figures 6D,E**). Two of five pigs in both the CV+PCV2b and CV+PCV2d group demonstrated moderate interstitial pneumonia in the lung tissues (**Figures 6F,G**). In CV+PCV2b pigs, two of five pigs had obvious microscopic lesions in SILN tissues (**Figure 6M**). Three of five pigs in CV+PCV2d pigs had obvious microscopic lesions in SILN tissues (**Figure 6N**). There were no obvious microscopic lesions in SILN or lung tissues collected at necropsy from the negative group pigs at 21 DPC (**Figures 6A,H**).

**Table 5:** Microscopic lesions and PCV2 antigens present in lymphoid tissues and lung at necropsy in the experimental pigs.

Group	No. of pigs testing positive/no. tested			
	
	Lymph nodes			Lung
		
	LD	HR	IHC	IP
VAC+PCV2b	0/5	0/5	1/5	1/5
VAC+PCV2d	2/5	1/5	1/5	2/5
CHAL+PCV2b	5/5	3/5	5/5	4/5
CHAL+PCV2d	4/5	2/5	4/5	4/5
CV+PCV2b	2/5	2/5	2/5	2/5
CV+PCV2d	3/5	2/5	2/5	2/5
Negative group	0/2	0/2	0/2	0/2


*LD, lymphoid depletion; HR, histiocytic replacement; IHC, immunohistochemistry; IP, interstitial pneumonia.*

### Amount of PCV2 Antigen in Tissues

**FIGURE 7:**
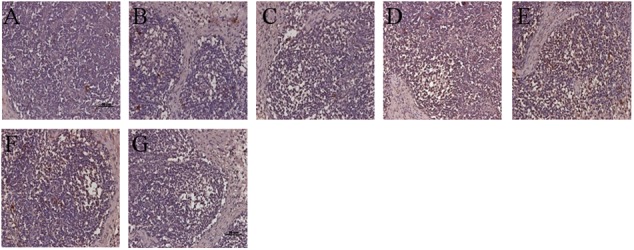
Immunohistochemistry detection of PCV2 antigen in SILN. **(A)** PCV2 antigen was not detected in lymph nodes of mock pigs. **(B)** Sparse staining for PCV2 antigen was detectable in the lymph nodes of VAC+PCV2b pigs. **(C)** Sparse staining for PCV2 antigen was detectable in lymph nodes of VAC+PCV2d pigs. **(D)** Strong staining for PCV2 antigen was evident in lymph nodes of CHAL+PCV2b pigs. **(E)** Strong staining for PCV2 antigen was evident in lymph nodes of CHAL+PCV2d pigs. **(F)** Sparse staining for PCV2 antigen was detectable in the lymph nodes of CV+PCV2b pigs. **(G)** Sparse staining for PCV2 antigen was detectable in lymph nodes of CV+PCV2d pigs.

Similar to the histological lesions, both the incidence and the amount of PCV2 antigen in SILN tissues were reduced in the VAC+PCV2b and VAC+PCV2d group pigs compared with unvaccinated but challenged animals. All five pigs in the CHAL+PCV2b group and four of five pigs in the CHAL+PCV2d group presented abundant PCV2 antigen in SILN tissues (**Figures 7D,E**), but only small amounts of PCV2 antigen were found in one pig in each of the VAC+PCV2b, VAC+PCV2d, CV+PCV2b, and CV+PCV2d groups (**Figures 7B,C,F,G**). PCV2 antigen in SILN tissues was 0 in negative group pigs (**Figure 7A**).

### Amount of PCV2 DNA in Tissues

**FIGURE 8:**
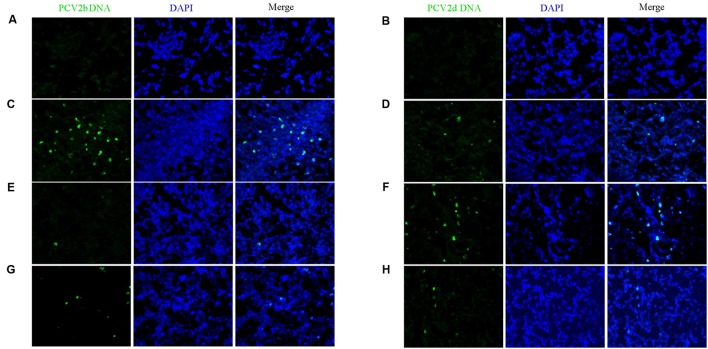
Fluorescence *in situ* hybridization detection of PCV2b and PCV2d DNA in SILN. **(A)** PCV2b DNA was not detected in lymph nodes of mock pigs. **(B)** PCV2d DNA was not detected in lymph nodes of mock pigs. **(C)** Strong staining for PCV2b DNA was evident in lymph nodes of CHAL+PCV2b pigs. **(D)** Sparse staining for PCV2b DNA was detectable in the lymph nodes of CV+PCV2b pigs. **(E)** Sparse staining for PCV2b DNA was detectable in the lymph nodes of VAC+PCV2b pigs. **(F)** Strong staining for PCV2d DNA was evident in lymph nodes of CHAL+PCV2d pigs. **(G)** Sparse staining for PCV2d DNA was detectable in lymph nodes of CV+PCV2d pigs. **(H)** Sparse staining for PCV2d DNA was detectable in lymph nodes of VAC+PCV2d pigs.

Fluorescence *in situ* hybridization (FISH) demonstrated that the amount of PCV2 DNA in SILN tissues were reduced in the VAC+PCV2b (**Figure 8E**), VAC+PCV2d (**Figure 8H**), CV+PCV2b (**Figure 8D**), and CV+PCV2d (**Figure 8G**) group pigs compared with unvaccinated but challenged animals (**Figures 8C,F**). All five pigs in the CHAL+PCV2b group and four of five pigs in the CHAL+PCV2d group presented abundant PCV2 DNA in SILN tissues (**Figures 8C,F**), but only small amounts of PCV2 DNA were found in one pig in each of the VAC+PCV2b (**Figure 8E**), VAC+PCV2d (**Figure 8H**), CV+PCV2b (**Figure 8D**), and CV+PCV2d (**Figure 8G**) groups. PCV2 DNA was 0 in negative group pigs (**Figures 8A,B**).

## Discussion

Porcine circovirus type 2 infection and the occurrence of PCVD remains a threat to the global pig industry. However, infection with PCV2 does not necessarily lead to disease. Concurrent or subsequent infections with other viral or bacterial agents is an important factor (Magar et al., 2000). Several studies have shown that PCV2b and PCV2d are the major genotypes prevalent in China and other countries (Xiao et al., 2015), and the PCV2 vaccine currently used is mainly based on the PCV2a/b inactivated vaccine. Although confirmed to be effective in preventing the occurrence of PCVD, there are still reports that vaccinated PCV2a vaccine pigs develop PCVD. Fenaux et al. (2003) constructed a chimeric PCV1-2 virus and subsequently confirmed that PCV1-2 maintained its parent PCV1 pathogenicity, but could induce both humoral and cellular immune responses, indicating that a chimeric PCV1-2 attenuated live vaccine could provide a comprehensive immune protection. Similarly, PCV1-2b constructed in our laboratory can also provide effective protection against infection with PCV2b (Li et al., 2015). In recent years, PCV2d became more and more prevalent in field cases all over the world, and was the predominant sub-genotype in South America, Europe, United States, Korea, and China so far (Xiao et al., 2015; Franzo et al., 2016; Kwon et al., 2017). Herein, we have evaluated the efficacy of chimeric PCV1-2b-induced protective and cross-protective immunity against PCV2b and PCV2d subtypes in pigs.

In this study, pigs vaccinated with chimeric PCV1-2b did not demonstrate elevated body temperature, diarrhea, weight loss, jaundice, anemia, respiratory disorders, or other PCVD-related clinical symptoms. PCV2 antibodies were detected by ELISA in the serum of vaccinated pigs following challenge and it was found that seroconversion occurred as early as 14 DPV, peaking at 28 DPV. After challenge, PCV2 antibody levels in the vaccinated group were slightly increased before attaining stable high levels.

Previous studies have shown that PCV1-2 chimeric virus DNA was detectable in pig serum after vaccination (Opriessnig et al., 2011). We found that chimeric PCV1-2b can also be detected in serum, and the PCV1-2b detection rate and content showed a downward trend at 28 DPV. Furthermore, real-time PCR results showed that low copy numbers of PCV1-2 nucleic acid could be detected in both nasal and fecal swabs of the vaccinated animals, indicating that vaccinated pigs may shed virus, but the number of PCV1-2 DNA copies is low. Therefore, PCV1-2 virus can successfully replicate in pigs, and thus continue to stimulate the body to produce antibodies against PCV2.

Studies have shown that vaccination with either the conventional PCV2 or PCV1-2 vaccines can effectively reduce viremia and lymph node PCV2 load in experimentally infected pigs, but cannot prevent PCV2 infection and shedding (Kixmoller et al., 2008; Opriessnig et al., 2011; Hemann et al., 2012). In this study, PCV2b and PCV2d DNA could be detected in the serum and swabs of vaccinated animals, but the number of PCV2 DNA copies was lower than in the unvaccinated but challenged groups. At 21 DPC, serum copies of PCV2 DNA from the vaccinated group were significantly lower than challenge-only piglets. Segales (2012) suggests that lymphocyte deletion, lymphocyte tissue infiltration, and PCV2 load in lymph nodes are important criteria for the diagnosis of PCVD and are important indicators of vaccine efficacy. At 21 DPC, PCV2 DNA was detectable in the SILN of all challenge-only piglets, but in only one piglet from the vaccinated group, and PCV2 DNA copies were significantly lower in this animal compared with the challenge-only group. Abundant PCV2b and PCV2d DNA was detected by FISH in the lymph nodes of unvaccinated infection-challenged pigs, compared with low levels of staining in the lymph nodes of vaccinated pigs. Additionally, abundant PCV2 antigen was detected by IHC in the lymph nodes of unvaccinated infection-challenged pigs, compared with low levels of staining in the lymph nodes of vaccinated pigs.

## Conclusion

In summary, our data suggest that the live-attenuated chimeric PCV1-2b vaccine can partially protect pigs from PCV2 viremia and significantly reduce the PCV2 viral load in lymph tissues, both of which are important factors in the pathogenesis of PCV2 infection. Therefore, the PCV1-2b vaccine virus can offer cross-protection against both PCV2b and PCV2d subtypes, which implied that the live-attenuated chimeric PCV1-2b vaccine was effective in prevention and control the current PCVAD caused by the two prevailing sub-genotype PCV2 strains. It may be advantageous in the future to combine the PCV1-2b vaccine from this study with the current PCV2 commercial vaccines for a more comprehensive protection.

## Ethics Statement

All experimental protocols were approved by the Animal Care and Use Committee of Yangzhou University [approval ID: SYXK (Su) 2005-0005]. All animal care and use protocols in this study were performed in accordance with the approved current guidelines.

## Author Contributions

CH, MF, and SG were participated in the research design. CH, MF, QC, WW, and XW were conducted the experiments. CH and QG were performed the data analysis. CH, MF, SG, and XL were wrote or contributed to the writing of the manuscript.

## Conflict of Interest Statement

The authors declare that the research was conducted in the absence of any commercial or financial relationships that could be construed as a potential conflict of interest.
